# Results from a cross-sectional sexual and reproductive health study among school girls in Tanzania: high prevalence of bacterial vaginosis

**DOI:** 10.1136/sextrans-2018-053680

**Published:** 2018-12-05

**Authors:** Suzanna C Francis, Christian Holm Hansen, Julia Irani, Aura Andreasen, Kathy Baisley, Vicky Jespers, Tania Crucitti, John Changalucha, Richard J Hayes, Soori Nnko, Deborah Watson-Jones, Anne Buvé

**Affiliations:** 1 London School of Hygiene and Tropical Medicine, London, UK; 2 MRC/UVRI and LSHTM Uganda Research Unit, Entebbe, Uganda; 3 Mwanza Intervention Trials Unit, National Institute for Medical Research, Mwanza, Tanzania; 4 Institute of Tropical Medicine, Antwerp, Belgium; 5 National Institute for Medical Research, Mwanza, Tanzania

**Keywords:** bacterial vaginosis, adolescent, Africa, women

## Abstract

**Objectives:**

Bacterial vaginosis (BV) increases women’s susceptibility to sexually transmitted infections (STIs) and HIV and may partly explain the high incidence of STI/HIV among girls and young women in East and southern Africa. The objectives of this study were to investigate the association between BV and sexual debut, to investigate other potential risk factors of BV and to estimate associations between BV and STIs.

**Methods:**

Secondary school girls in Mwanza, aged 17 and 18 years, were invited to join a cross-sectional study. Consenting participants were interviewed and samples were obtained for STI and BV testing. Factors associated with prevalent BV were analysed using multivariable logistic regression. Y-chromosome was tested as a biomarker for unprotected penile-vaginal sex.

**Results:**

Of the 386 girls who were enrolled, 163 (42%) reported having ever had penile-vaginal sex. Ninety-five (25%) girls had BV. The prevalence of BV was 33% and 19% among girls who reported or did not report having ever had penile-vaginal sex, respectively. BV was weakly associated with having ever had one sex partner (adjusted odds ratio (aOR) 1.59;95% CI 0.93 to 2.71) and strongly associated with two or more partners (aOR = 3.67; 95% CI 1.75 to 7.72), receptive oral sex (aOR 6.38; 95% CI 1.22 to 33.4) and having prevalent human papillomavirus infection (aOR = 1.73; 95% CI 1.02 to 2.95). Of the 223 girls who reported no penile-vaginal sex, 12 (5%) tested positive for an STI and 7 (3%) tested positive for Y-chromosome. Reclassifying these positive participants as having ever had sex did not change the key results.

**Conclusions:**

Tanzanian girls attending school had a high prevalence of BV. Increasing number of sex partner was associated with BV; however, 19% of girls who reported no penile-vaginal sex had BV. This suggests that penile-vaginal sexual exposure may not be a prerequisite for BV. There was evidence of under-reporting of sexual debut.

## Introduction

Globally, there are approximately 380 000 new HIV infections among girls and young women aged 10–24 years every year.^1^ In girls and young women in sub-Saharan Africa, the high incidence of HIV cannot be fully explained by behavioural factors alone (eg, frequent partner exchange).^2–4^ Higher per-partnership transmission probability could be due to biological factors evidenced by high rates of HIV positivity following the first few episodes of sexual intercourse after sexual debut.^3^ One factor that may increase susceptibility to HIV is the presence of abnormal vaginal microbiota, including bacterial vaginosis (BV).

BV is particularly prevalent in sub-Saharan Africa, with East African prevalences ranging from 21% to 34% among general population women.^5^ BV is associated with an increased risk of acquiring HIV infection in women.^6 7^ BV also increases the risk of other sexually transmitted infections (STIs) including human papillomavirus (HPV) and herpes simplex virus 2 (HSV-2) infection, which are in turn associated with HIV acquisition.^8–11^

The aetiology of BV is unclear, and the role of sexual transmission of BV-associated bacteria is an important unresolved question. Studies investigating BV around the time of sexual initiation may provide useful insight for understanding the aetiology of BV. Cross-sectional studies in the USA, Peru and Ecuador have documented BV among sexually naïve adolescents.^12 13^ Although a more recent cross-sectional study among young Australian women showed that BV was not detected among women without a history of sexual contact,^14^ a longitudinal study in the USA reported that colonisation with BV-associated bacteria was rare in sexually inexperienced women, apart from *Gardnerella vaginalis,* which was detected in 40% of participants at baseline. The initiation of penile-vaginal sex was associated with increased prevalence of *G. vaginalis*.^15^ Similarly, in a longitudinal study among school girls in Belgium, *G. vaginalis* and *Atopobium vaginae* were detected among some girls with no sexual experience (27% and 18%, respectively), and the initiation of sex was associated with increased presence of these BV-associated bacteria.^16^ The latter studies were carried out in populations with low BV and STI prevalence. More studies are needed to investigate BV among adolescent girls or young women around the time of their sexual debut in settings with high BV, STI and HIV prevalence. These studies should include other suggested risks for BV in the literature, including intravaginal practices, menstrual hygiene and tobacco use.^6 17 18^

We conducted a cross-sectional study among girls aged 17 and 18 years old attending secondary school in Mwanza, Tanzania to characterise the vaginal microbiota in girls who reported that they had never had sexual intercourse and girls who had passed their sexual debut. In this paper, we present the prevalence of BV, associated risk factors for BV and associations between BV and STIs. Previous research among girls in this region reported the median age of sexual debut to be 17 years.^19^

## Methods

### Study population and enrolment

Girls aged 16–18 enrolled in government-funded secondary day schools in Mwanza municipality were listed. Boarding schools were excluded because parents were not available to provide consent. Assuming that 50% of the girls reported sexual debut, a sample size of 400 was chosen to allow us to estimate the prevalence of BV with adequate precision in sexually active and sexually naïve girls. Recruitment was started in the schools with the highest numbers of pupils listed and continued till the sample size of 400 was reached.

Eligibility criteria included being aged 17 or 18 years old at enrolment, not having participated in a similar study, resident in Mwanza City and staying in Mwanza City for 1 month postenrolment to receive test results for STIs.

### Study procedures

Eligible girls were asked for their written informed consent/assent (with written informed consent from parents/guardians if the girls were aged 17 years or oral consent if girls were aged 18 years) before being enrolled and interviewed in a central research clinic. Structured face-to-face interviews obtained data on sociodemographics, hygiene practices, drug and alcohol use, sexual behaviours and symptoms of STIs. Urine and blood samples were collected. Five self-administered vaginal swabs were collected from non-pregnant girls. Girls who wanted to know their HIV status were provided with voluntary counselling and testing using on-site rapid tests; girls who did not want to know their status had HIV rapid tests performed in the central laboratory.

Girls with vaginal discharge syndrome, genital ulcer disease or pelvic inflammatory disease were offered free syndromic management on the same day. Laboratory results for treatable STIs and free treatment as required were provided to participants within 2 weeks.

### Laboratory methods

Laboratory testing was performed according to standard operating procedures. Urine samples were tested for pregnancy using the QuickVue+Test (QUIDEL, USA). Serum samples were used to test for IgG antibodies for HSV-2 by ELISA (Kalon Biological, UK). Syphilis was determined by the Immutrep Rapid Plasma Reagin test (Omega Diagnostics, Scotland) and the *Treponema pallidum* particle agglutination assay (SERODIA, Fujirebio, Japan).

All blood samples were screened with Determine HIV 1/2 rapid test (Alere, Japan). If reactive, they were tested with the Uni-Gold HIV rapid test (Trinity Biotech, Ireland). If both tests were reactive, the final result was deemed positive. If the Uni-Gold test was not reactive, the sample was tested with the HIV ½ Stat-Pak test (Chembio, USA). The final result was considered positive if the Stat-Pak result was reactive.

A vaginal swab was used to prepare a slide for Gram staining that was examined for vaginal yeast and for BV using the Nugent score.^20^ A Nugent score of 0–3 indicated normal microbiota, 4–6 indicated intermediate microbiota and 7–10 indicated BV. All slides were read by two trained technicians. In case of discordant results, the laboratory supervisor re-read the slide. The same swab was inoculated in an InPouch TV device (BioMed Diagnostics, USA) for *Trichomonas vaginalis*, incubated at 37°C, and read every other day for the presence of motile trichomonads for 5 days or until positive. Two flocked swabs (Copan, USA) were pooled and aliquots were tested for *Neisseria gonorrhoeae*, *Chlamydia trachomatis* and *Mycoplasma genitalium* by in-house real-time PCR.^21 22^ HPV genotyping was performed using the Roche Linear Array HPV Genotyping Test (Roche, USA), which detect 37 HPV genotypes.

Swabs from participants who denied having passed their sexual debut were tested for Y-chromosome by in-house real-time PCR as a biomarker for recent unprotected penile-vaginal sex.^23^

### Data management and statistical methods

Questionnaire data were double-entered into OpenClinica (Akaza Research, USA) and analysed using the SAS Software V.9.3 (SAS Institute, USA). We restricted the analyses to participants with Nugent score results.

Participant characteristics were examined by reported sexual debut status and by sociodemographic, behavioural and biological factors. Socioeconomic status (SES) was estimated using an indicator based on the type of possessions owned by the head of the household. BV prevalence was estimated separately for participants with and without a history of penile-vaginal sex. The infections investigated in this analysis were restricted to any positive HPV genotype, *T. vaginalis* and combined *C. trachomatis* and/or *N. gonorrhoeae* infection because of the relatively low prevalence of STIs found in this study.

We used logistic regression to estimate the effects of selected sociodemographic and behavioural risk factors on BV and summarised these in terms of crude and adjusted odds ratios (aORs) along with 95% CI and p values. The number and type of variables considered in this analysis were informed by causal associations found in the literature or potential confounders.

Using a hierarchical approach to the risk factor analysis, we first estimated the crude and independent effects of the sociodemographic characteristics on BV positivity (level 1). The independent effects were estimated using multivariable logistic regression adjusted for any other sociodemographic variables where p<0.10 in the adjusted analysis. This procedure was repeated for the analysis of behavioural risk factors (level 2), except that the multivariable model at this level was adjusted for other behavioural factors with adjusted associations with p<0.10 and the sociodemographic characteristics that were found to be independently associated with BV from the first stage of the analysis. Last, in a separate logistic regression analysis, we examined the associations between BV and prevalent STIs.

To investigate under-reporting of sexual behaviour, we carried out two sensitivity analyses. In the first analysis, we regrouped those participants who denied sexual activity but had a positive test for HSV-2, chlamydia, gonorrhoea, *M. genitalium* or Y-chromosome as sexually active and repeated the analysis described above. HPV and trichomoniasis were not included in this analysis as there is some evidence of non-sexual transmission for this infections,^24 25^ and HIV infection was not included as there was some evidence in the source notes that these were the result of vertical transmission. In the second sensitivity analysis, we used a more conservative approach and included all STIs (ie, including trichomoniasis, HPV and HIV) and Y chromosome.

## Results

We identified 26 non-boarding schools with at least 25 girls in the target age group for inclusion in the study; all but two participated in the study. Recruitment of girls took place between November 2013 and June 2014.

**Figure 1:**
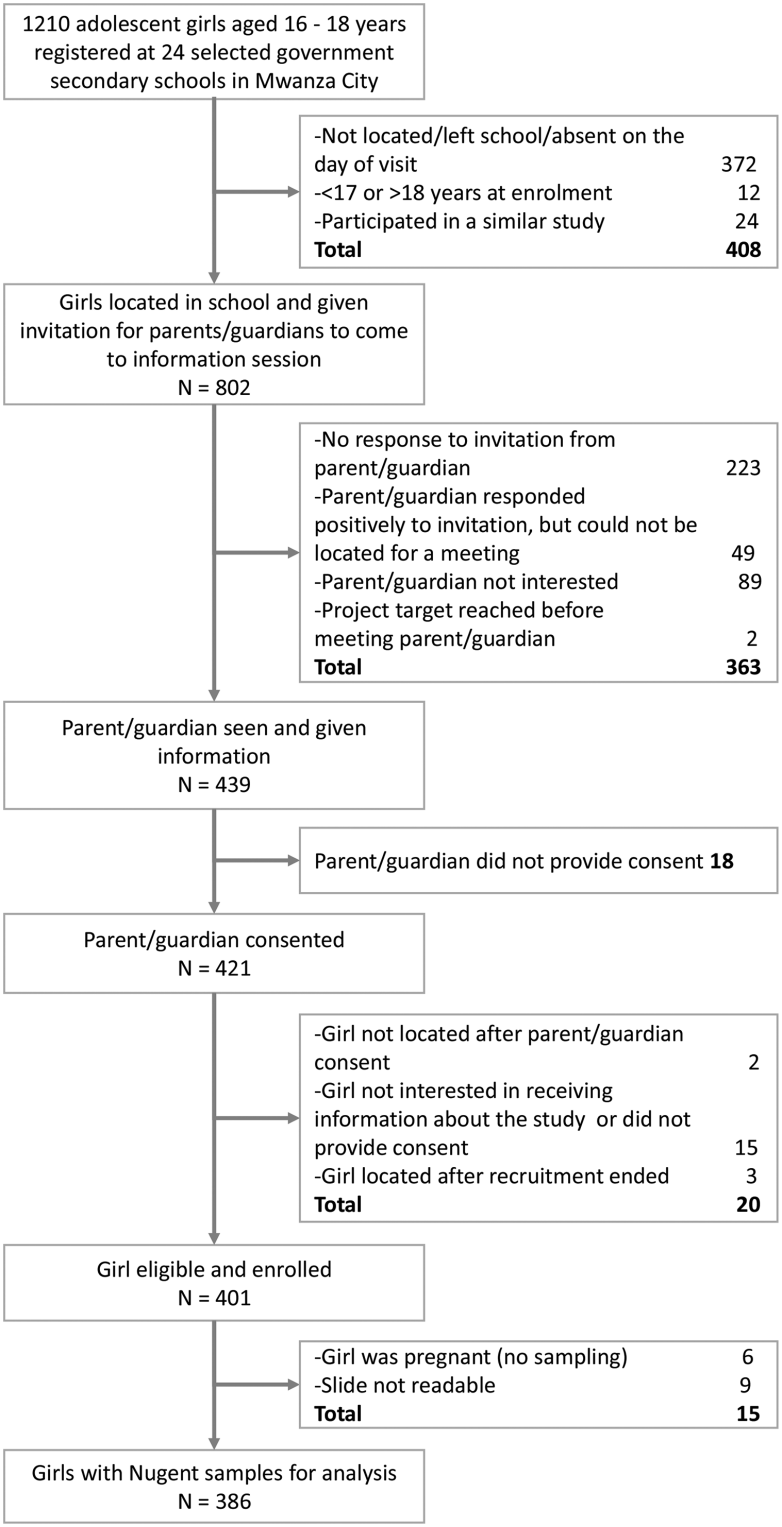
Recruitment procedure and derivation of the final analysis sample for 401 school girls enrolled in a cross-sectional study in Mwanza, Tanzania.

There were 1210 girls registered at the 24 participating schools who were aged 17 or 18 years (figure 1). Of these, 802 (66%) girls were successfully contacted at school and their parents were invited for a study information meeting and 55% (439) of parents attended. A total of 401 (95%) girls provided consent/assent and were enrolled in the study. Of these, 386 (96%) had a BV result and were included in the analyses.

### Participant characteristics

Over half of the participants were 17 years old (56%, table 1) and 75% were born in Mwanza region. Most girls lived in a household with at least their mothers (63%), but a substantial proportion reported not living with either of their parents (30%). Few girls reported spending at least one night away from home in the last 3 months (12%) or having ever drunk alcohol (3%). None reported smoking cigarettes or using illicit drugs.

**Table 1:** Sociodemographic characteristics, reported sexual history and hygiene management and reproductive tract infections among adolescent school girls in Mwanza City, Tanzania

	Participant reported having had penile-vaginal sex				All	
	Yes		No			
**Total**	163	42%	223	58%	386	100%
**Sociodemographic characteristics**						
Age (years)						
17	80	49%	135	61%	215	56%
18	83	51%	88	39%	171	44%
Born in						
Mwanza region	118	72%	172	77%	290	75%
Other region	45	28%	51	23%	96	25%
Lives with						
Mother (+/-father/other person)	98	60%	147	66%	245	63%
Father (+/-other person, but not mother)	12	7%	12	5%	24	6%
Does not live with mother or father	53	33%	64	29%	117	30%
Number of people in household						
1–5	69	42%	65	29%	134	35%
6–7	46	28%	84	38%	130	34%
8 or more	48	29%	74	33%	122	32%
SES indicator (possessions)						
Car	8	5%	16	7%	24	6%
TV, but no car	76	47%	89	40%	165	43%
Cell phone, no car or TV	73	45%	110	49%	183	47%
None of the above	6	4%	8	4%	14	4%
Nights outside home, last 3 months						
None	138	85%	202	91%	340	88%
One or more	25	15%	21	9%	46	12%
**Hygiene behaviour**						
Menstrual hygiene management						
Reusable cloth	60	37%	90	40%	150	39%
Underpants	161	99%	211	95%	372	96%
Sanitary pads	133	82%	149	67%	282	73%
Tampons or toilet paper	1	1%	2	1%	3	1%
Intravaginal cleansing						
No cleansing	121	74%	207	93%	328	85%
Plain water	23	14%	11	5%	34	9%
Soap	17	10%	5	2%	22	6%
Cloth, cotton wool, detergents	2	1%	0	0%	2	1%
Method of cleaning after defecation						
Water only	149	91%	193	87%	342	89%
Toilet paper	9	6%	22	10%	31	8%
Other	5	3%	8	4%	13	3%
Direction of cleaning after defecation						
Front to back	118	72%	176	79%	294	76%
Back to front	45	28%	47	21%	92	24%
**Sexual behaviour**						
Touched penis with hands	19	12%	3	1%	22	6%
Man/boy touched vagina with hands	34	21%	1	0%	35	9%
Had penis in her mouth	2	1%	1	0%	3	1%
Receptive oral sex	8	5%	1	0%	9	2%
Penis rubbed against her genitals (without penetration)	9	6%	0	0%	9	2%
Anal sex*	2	1%	0	0%	2	1%
Number of life-time (penile-vaginal sex) partners						
One	123	75%				
Two	31	19%				
Three or more	9	6%				
Age of first sexual partner						
<1 year older	16	10%				
1–2 years older	31	19%				
2–3 years older	30	18%				
>3 years older	64	39%				
Don’t know/no answer	22	13%				
Frequency of condom use with current/latest partner						
Never	67	41%				
Some of the time	24	15%				
Always	69	42%				
Don’t know/no answer	3	2%				
**BV, vaginal yeast and sexually transmitted infections**						
BV (Nugent 7–10)	53	33%	42	19%	95	25%
Intermediate (Nugent 4–6)	17	10%	12	5%	29	7%
Vaginal yeast	10	6%	11	5%	21	5%
*Chlamydia trachomatis*	8	5%	1	0%	9	2%
*Neisseria gonorrhoeae*	4	2%	4	2%	8	2%
*Trichomonas vaginalis*	15	9%	2	1%	17	4%
*Mycoplasma genitalium*	6	4%	3	1%	9	2%
Active syphilis	0	0%	0	0%	0	0%
Human papillomavirus—any genotype†	84	52%	41	18%	125	33%
Herpes simplex virus-2	5	3%	4	2%	9	2%
HIV†	0	0%	3	1%	3	1%

*Missing data for one participant.

†Missing data for two participants.

BV, bacterial vaginosis; SES, socioeconomic status.

All girls in the study had passed menarche, and the median age of menarche was 14 (IQR 14–15). Girls reported using multiple types of menstrual hygiene products; the most common included underpants (96%), commercial sanitary pads (73%) and reusable cloth (39%). Only 15% reported intravaginal cleansing and this was more common among girls reporting penile-vaginal sex 26% vs 7%. Only one girl reported intravaginal insertion (insertion of lemon or lime juice). Almost all girls reported washing with water after defecation (99.5%); a few used toilet paper (8%) or another material (eg, cloth or leaves). Overall, 24% reported washing or wiping their vulva/perianal area from back to front after defecation.

All girls were unmarried. Of the 386 participants, 6% reported having touched a penis, 9% reported that a man or boy had touched her genitals, 1% reported having provided oral sex, 2% reported ever receiving oral sex from a male partner, 2% reported having a penis rub her genitals without penetration, 1% reported anal sex and 42% reported having penile-vaginal sex with at least one sex partner. Of those who reported penile-vaginal sex, 25% had more than one partner and 42% reported always using condoms with their last partner. There were only three girls who reported non-coital sexual exposures without penile-vaginal penetrative sex.

### Prevalence of BV, vaginal yeast and STIs

The overall prevalence of BV was 25% (95/386), with 19% in girls who reported no penile-vaginal sex and 33% in girls reporting having had penile-vaginal sex (p=0.002, Pearson χ² test).

The prevalence of vaginal yeast was 5%. The overall HPV prevalence was 33% (125/384). The HPV prevalence among girls who reported and did not report penile-vaginal sex was 52% and 18%, respectively. The prevalence of *T. vaginalis* infection was 4%, and the prevalences of *C. trachomatis*, *N. gonorrhoeae*, *M. genitalium* were all 2%. HSV-2 seroprevalence was 2%, and HIV seroprevalence was 1%. There were no positive tests for syphilis.

### Sexual debut and other potential risk factors associated with prevalent BV

Results from the analysis of sociodemographic and behavioural risk factors for BV are shown in table 2. There was modest evidence that lower SES was associated with BV. No other sociodemographic factors were associated with BV.

**Table 2:** Prevalence of BV and associations with sociodemographic and behavioural determinants among adolescent school girls in Mwanza City, Tanzania

	N	BV n (%)	OR (95% CI)	P values	Adjusted OR (95% CI)§	P values
Total	386	95 (25)	–	–	–	–
**Sociodemographic factors**						
Age (years)						
17	215	55 (26)	1	0.620	1	0.642
18	171	40 (23)	0.88 (0.56 to 1.42)		0.89 (0.56 to 1.43)	
SES indicator (possessions)*						
Car in household	24	2 (8)	0.70 (0.49 to 1.01)	0.054	0.71 (0.49 to 1.01)	0.054
TV, but no car in household	165	39 (24)	–		–	
Cell phone, no car or TV in household	183	49 (27)	–		–	
None of the above	14	5 (36)	–		–	
Lives with						
Mother (+/-father/other person)	245	58 (24)	1	0.843	1	0.564
Father (+/-other person, but not mother)	24	6 (25)	1.08 (0.41 to 2.83)		1.13 (0.42 to 3.00)	
Does not live with mother or father	117	31 (26)	1.16 (0.70 to 1.93)		1.33 (0.79 to 2.25)	
**Behavioural factors**						
Menstrual hygiene management†						
Sanitary pads or towels	203	52 (26)	1	0.719	1	0.675
Cloth, toilet paper or pants	179	43 (24)	0.92 (0.58 to 1.46)		0.90 (0.55 to 1.47)	
Intravaginal cleansing						
No cleansing	328	79 (24)	1	0.594	1	0.565
Using water only	34	8 (24)	0.97 (0.42 to 2.23)		0.60 (0.24 to 1.53)	
Using other substances	24	8 (33)	1.58 (0.65 to 3.82)		0.91 (0.33 to 2.47)	
Direction of cleaning after defecation						
Front to back	294	72 (24)	1	0.921	1	0.906
Back to front	92	23 (25)	1.03 (0.60 to 1.77)		1.04 (0.59 to 1.82)	
Man/boy touched vagina with hands						
No	351	81 (23)	1	0.030	1	0.512
Yes	35	14 (40)	2.22 (1.08 to 4.57)		0.73 (0.29 to 1.85)	
Receptive oral sex						
No	377	88 (23)	1	0.003	1	0.028
Yes	9	7 (78)	11.5 (2.35 to 56.3)		6.38 (1.22 to 33.4)	
Life-time sexual partners						
None	223	42 (19)	1	<0.001	1	0.002
One	123	33 (27)	1.58 (0.94 to 2.66)		1.59 (0.93 to 2.71)	
Two or more	40	20 (50)	4.31 (2.13 to 8.72)		3.67 (1.75 to 7.72)	
Condom use with latest partner†,‡						
Always	69	19 (28)	1	0.306	1	0.553
Not always	91	32 (35)	1.43 (0.72 to 2.82)		1.25 (0.60 to 2.57)	
Age of first sexual partner†,‡						
<1 year older	16	5 (31)	1	0.350	1	0.371
1–2 years older	31	6 (19)	0.53 (0.13 to 2.10)		0.44 (0.10 to 1.88)	
2–3 years older	30	11 (37)	1.27 (0.35 to 4.64)		1.22 (0.31 to 4.76)	
3 or more years older	64	24 (38)	1.32 (0.41 to 4.26)		1.07 (0.32 to 3.67)	

*SES indicator was fitted as a continuous covariate; the OR of 0.70 estimates the decrease in odds of BV for a one-step increase in SES score.

†Missing data for some participants.

‡Analysis restricted to those who reported having had at least one partner.

§All were adjusted for age and SES; behavioural factors were also adjusted for lifetime sexual partners and oral sex.

BV, bacterial vaginosis (Nugent score ≥7); SES, socioeconomic status.

The number of life-time sexual partners was independently associated with BV (p=0.002) and participants reporting two or more sexual partners had 3.67 (95% CI 1.75 to 7.72 times the odds of BV compared with those who reported no past (penile-vaginal) sex partners. In addition, a higher proportion of girls who reported receiving oral sex from a male partner had BV 78% vs 23%), although numbers were low and CIs were wide (aOR=6.38; 95% CI 1.22 to 33.4). No other behaviours were independently associated with BV.

### Associations between BV and other reproductive tract infections including STIs

HPV infection was independently associated with BV (aOR=1.73; 95% CI 1.02 to 2.95, table 3). Vaginal yeast, chlamydial and/or gonorrhoea (combined) and trichomoniasis were not associated with BV.

**Table 3:** Associations between BV and sexually transmitted infections among adolescent school girls in Mwanza City, Tanzania

	N	BV n (%)	OR (95% CI)	P values	Adjusted OR (95% CI)†	P values
Total	386	95 (25)	–	–	–	–
Vaginal yeast						
Negative	365	92 (25)	1	0.268	1	0.336
Positive	21	3 (14)	0.50 (0.14 to 1.72)		0.54 (0.15 to 1.91)	
*Chlamydia trachomatis* or *Neisseria gonorrhoea*						
Negative	369	91 (25)	1	0.916	1	0.542
Positive	17	4 (24)	0.94 (0.30 to 2.96)		0.68 (0.20 to 2.33)	
*Trichomonas vaginalis*						
Negative	369	89 (24)	1	0.300	1	0.935
Positive	17	6 (35)	1.72 (0.62 to 4.78)		0.95 (0.31 to 2.92)	
Human papillomavirus*						
Negative	259	50 (19)	1	<0.001	1	0.042
Positive for any genotype	125	45 (36)	2.35 (1.46 to 3.79)		1.73 (1.02 to 2.95)	

*Data missing for two participants in the BV negative group.

†All adjusted for age, SES, lifetime sexual partners, receptive oral sex and HPV.

BV, bacterial vaginosis (Nugent score ≥7); HPV, human papillomavirus; SES, socioeconomic status.

### Sensitivity analysis

A total 19 (9%) of the 223 participants who reported no penile-vaginal sex, tested positive for HSV-2, chlamydia, gonorrhoea, *M. genitalium* (n=12) or Y-chromosome (n=7). For the second sensitivity analysis, 56 (25%) tested positive for any STI (including HPV, trichomoniasis and HIV) and Y-chromosome. Positive tests were reclassified as having had sex for the first and second sensitivity analyses. Using these classifications, the prevalence of BV among those who had not passed their sexual debut was 20% (40/204) and 16% (26/167) in the first and second sensitivity analyses, respectively. The estimated ORs were similar to those derived from the main analysis (online supplementary material 1).

10.1136/sextrans-2018-053680.supp1 Supplementary data

## Discussion

We found a high prevalence of BV among girls around the age or expected age of sexual debut. Increasing number of lifetime partners was strongly associated with BV. However, a substantial proportion of girls who denied penile-vaginal intercourse were diagnosed with BV. To our knowledge, this is the first paper to investigate BV among girls or young women in sub-Saharan Africa around their sexual debut. Given the known association between BV and HIV, and the high burden of HIV among girls and young women, the high prevalence of BV is of public health concern.

The strong associations between BV, number of lifetime sex partners, oral sex and HPV are consistent with the literature. Fethers and colleagues found an association between new or multiple partners and BV in a meta-analysis (pooled RR:1.6; 95% CI 1.5 to 1.8).^26^ Several studies report an association between receiving oral sex and BV.^14 27 28^ While the literature shows strong associations between BV and prevalent bacterial and viral STIs,^8–11^ we only saw an association with prevalent HPV infection. However, this may be due to the low prevalence of other STIs in this study.

Our study strongly suggests that penile-vaginal sex increases the risk for BV; however, there is also evidence that it may not be a prerequisite for BV. This is consistent with past cross-sectional studies which reported BV among sexually naïve adolescents.^12 13^ More recent studies in populations with low BV prevalence have shown that BV has not been detected among women without a history of coital or non-coital sexual contact.^14 15^ In our study, only 3 (1%) girls who reported not having penile-vaginal sex, reported non-coital sexual exposure. While our results imply that girls and young women in sub-Saharan Africa may experience BV prior to sexual debut, these results must be considered in the context of under-reporting of sexual behaviours. We found several cases of STIs and detected Y-chromosome among girls who did not report penile-vaginal sex. Under-reporting of sexual behaviour among adolescents has been well documented internationally especially during face-to-face interviews^29^ and has also been documented in Tanzania.^25 30^ Although the study staff emphasised confidentiality of results to both parents and participants, students may have feared stigmatisation, school expulsion and physical punishment.^25^ Additionally, we did not ask about female sexual contacts or sexual assault among those girls who denied penile-vaginal sex. We carried out two sensitivity analyses to address reporting bias by reclassifying those in the sexually naïve group with STIs or Y-chromosome and found no substantial differences in the results in both analyses. However, this approach is unsatisfactory as it only redresses a proportion of the reporting bias. Reporting bias of sexual behaviour represents major impediment for the fields of adolescent health and HIV/STI prevention. Better methods of estimating reporting bias for sensitive behavioural data are needed. In addition, longitudinally designed research is needed to better understand changes in the vaginal microbiota before and after sexual debut.

The overall HPV prevalence was high (33%), with 52% and 18% prevalence among girls reporting and not reporting penile-vaginal sex, respectively. Houlihan and colleagues reported an overall HPV prevalence of 8% among girls who reported never having penile-vaginal sex and 32% among girls who had passed sexual debut in a similar population in Mwanza Tanzania.^25^ Conversely, the HSV-2 prevalence was lower than expected. Past studies from this region using the same laboratory test have shown higher prevalence of HSV-2, increasing rapidly after sexual debut; in 1999, Obasi and colleagues found an HSV-2 prevalence of 18%, 22% and 33% among 15, 16 and 17 year olds, respectively.^31^

This study had several strengths. We asked detailed information about behaviour and experiences of first sex and were able to measure STIs and HIV among girls recruited from a school setting. However, there were several limitations in addition to the issue of reporting bias noted above. This is a cross-sectional study and, therefore, the direction of causality cannot be discerned, most relevant for the association between BV and STIs. Also, we enrolled 401 girls from a list of 1210 (34%), which suggests possible selection bias. Most of the girls not enrolled were either not found at school or the parent was not available or willing to provide consent—these girls may be at higher risk for STI and BV, therefore we may have underestimated the prevalence of STIs and BV.

In conclusion, a high prevalence of BV and HPV was found among adolescent girls attending secondary schools in Tanzania. Penile-vaginal sex and increasing number of partners appear to increase the risk for BV, yet there is some evidence that girls may be entering sexual debut with altered vaginal microbiota, placing them at risk for HIV. Importantly, there was strong evidence of under-reporting of sexual activity in this population, and further methodological studies are needed to investigate improved reporting for sexual behaviour in adolescent populations. Longitudinal studies are needed to better understand the dynamics of vaginal microbiota during this crucial time of sexual transition in high HIV prevalence settings.

**Key messages**

- Tanzanian girls attending school had a high prevalence of bacterial vaginosis (BV) (25%).
- Sexual debut and increasing number of sex partners was associated with BV; however, 19% of girls who reported no penile-vaginal sex had BV.
- There was strong evidence of under-reporting of sexual activity in this population, and further methodological studies are needed to investigate improved reporting for sexual behaviour in adolescent populations.
- Longitudinal studies are needed to better understand the dynamics of vaginal microbiota during this crucial time of sexual transition in high HIV prevalence settings.
