# Supplementary material for: Results from a cross-sectional sexual and reproductive health study among school girls in Tanzania: high prevalence of bacterial vaginosis
Source: Sex Transm Infect. 2018 Dec 5;95(3):219–27. doi: 10.1136/sextrans-2018-053680 (PMC6580744; doi:10.1136/sextrans-2018-053680)
Supplement: Supplementary data [file sextrans-2018-053680supp001.docx]

Results from a cross-sectional sexual and reproductive health study among school girls in Tanzania: high prevalence of bacterial vaginosis.

Francis SC^1^, Holm Hansen C^1,2^, Irani J^3,4^, Andreasen A^1,3^, Baisley K^1^, Jespers V^4^, Crucitti T^4^, Changalucha C^5^, Hayes RJ^1^, Nnko S^5^, Watson-Jones D^1,3^, Buve A^4^

^1^London School of Hygiene and Tropical Medicine, London, UK

^2^ MRC/UVRI and LSHTM Uganda Research Unit, Entebbe, Uganda

^3^Mwanza Intervention Trials Unit, National Institute for Medical Research, Mwanza, Tanzania

^4^Institute of Tropical Medicine, Antwerp, Belgium

^5^National Institute for Medical Research, Mwanza, Tanzania

Contents

Sensitivity analysis 1: Participants who tested positive for Y-chromosome, HSV-2, chlamydia, gonorrhoea and *M. genitalium* are assumed to have had penile-vaginal sex irrespective of self-reported sexual history.

Table S1.1 First sensitivity analysis of prevalence of BV and associations with sociodemographic and behavioural determinants among adolescent schoolgirls in Mwanza city, Tanzania.

Table S1.2 First sensitivity analysis of BV and associations with sexually transmitted infections among adolescent schoolgirls in Mwanza city, Tanzania.

Sensitivity analysis 2: Participants who tested positive for Y-chromosome, HSV-2, chlamydia, gonorrhoea, *M. genitalium,* trichomoniasis, HPV and HIV are assumed to have had penile-vaginal sex irrespective of self-reported sexual history

Table S2.1 Second sensitivity analysis of prevalence of BV and associations with sociodemographic and behavioural determinants among adolescent schoolgirls in Mwanza city, Tanzania.

Table S2.2 Second sensitivity analysis of BV and associations with sexually transmitted infections among adolescent schoolgirls in Mwanza city, Tanzania.

| Table S1.1 First sensitivity analysis of prevalence of BV and associations with sociodemographic and behavioural determinants among adolescent schoolgirls in Mwanza city, Tanzania. ^a^ | | | | | | |
| --- | --- | --- | --- | --- | --- | --- |
|  | **N** | **BV**  **n (%)** | **Odds ratio**  **(95% CI)** | **p** | **Adjusted odds ratio (95% CI)** ^e^ | **p** |
| **Total** | 386 | 95 (25) | - | - | - | - |
| **SOCIO-DEMOGRAPHIC FACTORS** | |  |  |  |  |  |
| **Age (years)** |  |  |  |  |  |  |
| 17 | 215 | 55 (26) | 1 | 0.620 | 1 | 0.642 |
| 18 | 171 | 40 (23) | 0.88 (0.56 to 1.42) |  | 0.89 (0.56 to 1.43) |  |
| **SES indicator (possessions)** ^b^ |  |  |  |  |  |  |
| Car | 24 | 2 ( 8) | 0.70 (0.49 to 1.01) | 0.054 | 0.71 (0.49 to 1.01) | 0.054 |
| TV, but no car | 165 | 39 (24) | - |  | - |  |
| Cell phone, no car or TV | 183 | 49 (27) | - |  | - |  |
| None of the above | 14 | 5 (36) | - |  | - |  |
| **Lives with** |  |  |  |  |  |  |
| Mother | 245 | 58 (24) | 1 | 0.843 | 1 | 0.564 |
| Father (but not mother) | 24 | 6 (25) | 1.08 (0.41 to 2.83) |  | 1.13 (0.42 to 3.00) |  |
| Does not live with parents | 117 | 31 (26) | 1.16 (0.70 to 1.93) |  | 1.33 (0.79 to 2.25) |  |
| **BEHAVIOURAL FACTORS** |  |  |  |  |  |  |
| **Menstrual hygiene management** ^c^ |  |  |  |  |  |  |
| Sanitary pads or towels | 203 | 52 (26) | 1 | 0.719 | 1 | 0.623 |
| Cloth, toilet paper or pants | 179 | 43 (24) | 0.92 (0.58 to 1.46) |  | 0.88 (0.54 to 1.45) |  |
| **Intravaginal cleansing** |  |  |  |  |  |  |
| No cleansing | 328 | 79 (24) | 1 | 0.594 | 1 | 0.621 |
| Using water only | 34 | 8 (24) | 0.97 (0.42 to 2.23) |  | 0.63 (0.25 to 1.60) |  |
| Using other substances | 24 | 8 (33) | 1.58 (0.65 to 3.82) |  | 0.94 (0.35 to 2.58) |  |
| **Direction of cleaning after defecation** |  |  |  |  |  |  |
| Front to back | 294 | 72 (24) | 1 | 0.921 | 1 | 0.832 |
| Back to front | 92 | 23 (25) | 1.03 (0.60 to 1.77) |  | 1.06 (0.61 to 1.86) |  |
| **Man/boy touched vagina with hands** |  |  |  |  |  |  |
| No | 351 | 81 (23) | 1 | 0.030 | 1 | 0.621 |
| Yes | 35 | 14 (40) | 2.22 (1.08 to 4.57) |  | 0.79 (0.31 to 2.00) |  |
| **Receptive oral sex** |  |  |  |  |  |  |
| No | 377 | 88 (23) | 1 | 0.003 | 1 | 0.024 |
| Yes | 9 | 7 (78) | 11.5 (2.35 to 56.3) |  | 6.68 (1.28 to 34.9) |  |
| **Life-time sexual partners** |  |  |  |  |  |  |
| **None** | **204** | **40 (20)** | **1** | **<0.001** | **1** | **0.005** |
| **One** | **142** | **35 (25)** | **1.34 (0.80 to 2.44)** |  | **1.34 (0.79 to 2.27)** |  |
| **Two or more** | **40** | **20 (50)** | **4.10 (2.02 to 8.34)** |  | **3.48 (1.65 to 7.35)** |  |
| **Condom use with latest partner** ^c d^ |  |  |  |  |  |  |
| Always | 69 | 19 (28) | 1 | 0.306 | 1 | 0.553 |
| Not always | 91 | 32 (35) | 1.43 (0.72 to 2.82) |  | 1.25 (0.60 to 2.57) |  |
| **Age of first sexual partner** ^c d^ |  |  |  |  |  |  |
| < 1 year older | 16 | 5 (31) | 1 | 0.350 | 1 | 0.371 |
| 1-2 years older | 31 | 6 (19) | 0.53 (0.13 to 2.10) |  | 0.44 (0.10 to 1.88) |  |
| 2-3 years older | 30 | 11 (37) | 1.27 (0.35 to 4.64) |  | 1.22 (0.31 to 4.76) |  |
| 3 or more years older | 64 | 24 (38) | 1.32 (0.41 to 4.26) |  | 1.07 (0.32 to 3.67) |  |
|  |  |  |  |  |  |  |

BV=Bacterial vaginosis (Nugent score ≥7). SES=Socio-economic status. ^a^ Sensitivity analysis assuming participants with Y-chromosome and STIs have all had penile-vaginal sex irrespective of self-reported sexual history. ^b^ SES indicator was fitted as a continuous covariate; the odds ratio of 0.70 estimates the decrease in odds of BV for a one-step increase in SES score. ^c^ Missing data for some participants. ^d^ Analysis restricted to those who reported having had at least one partner. ^e^ All were adjusted for age and SES; behavioural factors were also adjusted for lifetime sexual partners and oral sex.

| Table S1.2. First sensitivity analysis of BV and associations with sexually transmitted infections among adolescent schoolgirls in Mwanza city, Tanzania.^a^ | | | | | | |
| --- | --- | --- | --- | --- | --- | --- |
|  | **N** | **BV**  **n (%)** | **Odds ratio**  **(95% CI)** | **p** | **Adjusted odds ratio (95% CI)**^c^ | **p** |
| **Total** | 386 | 95 (25) | - | - | - | - |
| **Vaginal yeast** |  |  |  |  |  |  |
| Negative | 365 | 92 (25) | 1 | 0.268 | 1 | 0.330 |
| Positive | 21 | 3 (14) | 0.50 (0.14 to 1.72) |  | 0.53 (0.15 to 1.90) |  |
| **Chlamydia or gonorrhoea** |  |  |  |  |  |  |
| Negative | 369 | 91 (25) | 1 | 0.916 | 1 | 0.549 |
| Positive | 17 | 4 (24) | 0.94 (0.30 to 2.96) |  | 0.69 (0.20 to 2.36) |  |
| **Trichomonas vaginalis** |  |  |  |  |  |  |
| Negative | 369 | 89 (24) | 1 | 0.300 | 1 | 0.983 |
| Positive | 17 | 6 (35) | 1.72 (0.62 to 4.78) |  | 1.01 (0.33 to 3.09) |  |
| **Human papillomavirus** ^b^ |  |  |  |  |  |  |
| Negative | 259 | 50 (19) | 1 | <0.001 | 1 | 0.028 |
| Positive | 125 | 45 (36) | 2.35 (1.46 to 3.79) |  | 1.83 (1.07 to 3.13) |  |

BV=Bacterial vaginosis (Nugent score ≥7). ^a^ Sensitivity analysis assuming participants with Y-chromosome and STIs have all had penile-vaginal sex irrespective of self-reported sexual history. ^b^ Data missing for two participants in the BV negative group. ^c^ All adjusted for age, SES, lifetime sexual partners, oral sex and HPV.

| Table S2.1. Second sensitivity analysis of prevalence of BV and associations with sociodemographic and behavioural determinants among adolescent schoolgirls in Mwanza city, Tanzania. ^a^ | | | | | | |
| --- | --- | --- | --- | --- | --- | --- |
|  | **N** | **BV**  **n (%)** | **Odds ratio**  **(95% CI)** | **P** | **Adjusted odds ratio (95% CI)** ^e^ | **p** |
| **Total** | 386 | 95 (25) | - | - | - | - |
| **SOCIO-DEMOGRAPHIC FACTORS** | |  |  |  |  |  |
| **Age (years)** |  |  |  |  |  |  |
| 17 | 215 | 55 (26) | 1 | 0.620 | 1 | 0.642 |
| 18 | 171 | 40 (23) | 0.88 (0.56 to 1.42) |  | 0.89 (0.56 to 1.43) |  |
| **SES indicator (possessions)** ^b^ |  |  |  |  |  |  |
| Car | 24 | 2 ( 8) | 0.70 (0.49 to 1.01) | 0.054 | 0.71 (0.49 to 1.01) | 0.054 |
| TV, but no car | 165 | 39 (24) | - |  | - |  |
| Cell phone, no car or TV | 183 | 49 (27) | - |  | - |  |
| None of the above | 14 | 5 (36) | - |  | - |  |
| **Lives with** |  |  |  |  |  |  |
| Mother | 245 | 58 (24) | 1 | 0.843 | 1 | 0.564 |
| Father (but not mother) | 24 | 6 (25) | 1.08 (0.41 to 2.83) |  | 1.13 (0.42 to 3.00) |  |
| Does not live with parents | 117 | 31 (26) | 1.16 (0.70 to 1.93) |  | 1.33 (0.79 to 2.25) |  |
| **BEHAVIOURAL FACTORS** |  |  |  |  |  |  |
| **Menstrual hygiene management** ^c^ |  |  |  |  |  |  |
| Sanitary pads or towels | 203 | 52 (26) | 1 | 0.719 | 1 | 0.685 |
| Cloth, toilet paper or pants | 179 | 43 (24) | 0.92 (0.58 to 1.46) |  | 0.90 (0.55 to 1.48) |  |
| **Intravaginal cleansing** |  |  |  |  |  |  |
| No cleansing | 328 | 79 (24) | 1 | 0.594 | 1 | 0.461 |
| Using water only | 34 | 8 (24) | 0.97 (0.42 to 2.23) |  | 0.56 (0.22 to 1.42) |  |
| Using other substances | 24 | 8 (33) | 1.58 (0.65 to 3.82) |  | 0.84 (0.31 to 2.28) |  |
| **Direction of cleaning after defecation** |  |  |  |  |  |  |
| Front to back | 294 | 72 (24) | 1 | 0.921 | 1 | 0.985 |
| Back to front | 92 | 23 (25) | 1.03 (0.60 to 1.77) |  | 1.01 (0.57 to 1.77) |  |
| **Man/boy touched vagina with hands** |  |  |  |  |  |  |
| No | 351 | 81 (23) | 1 | 0.030 | 1 | 0.465 |
| Yes | 35 | 14 (40) | 2.22 (1.08 to 4.57) |  | 0.71 (0.29 to 1.77) |  |
| **Receptive oral sex** |  |  |  |  |  |  |
| No | 377 | 88 (23) | 1 | 0.003 | 1 | 0.026 |
| Yes | 9 | 7 (78) | 11.5 (2.35 to 56.3) |  | 6.59 (1.25 to 34.7) |  |
| **Life-time sexual partners** |  |  |  |  |  |  |
| **None** | **167** | **26 (16)** | **1** | **<0.001** | **1** | **0.004** |
| **One** | **179** | **49 (27)** | **2.04 (1.20 to 3.48)** |  | **2.07 (1.21 to 3.55)** |  |
| **Two or more** | **40** | **20 (50)** | **5.42 (2.57 to 11.5)** |  | **4.65 (2.12 to 10.2)** |  |
| **Condom use with latest partner** ^c d^ |  |  |  |  |  |  |
| Always | 69 | 19 (28) | 1 | 0.306 | 1 | 0.553 |
| Not always | 91 | 32 (35) | 1.43 (0.72 to 2.82) |  | 1.25 (0.60 to 2.57) |  |
| **Age of first sexual partner** ^c d^ |  |  |  |  |  |  |
| < 1 year older | 16 | 5 (31) | 1 | 0.350 | 1 | 0.371 |
| 1-2 years older | 31 | 6 (19) | 0.53 (0.13 to 2.10) |  | 0.44 (0.10 to 1.88) |  |
| 2-3 years older | 30 | 11 (37) | 1.27 (0.35 to 4.64) |  | 1.22 (0.31 to 4.76) |  |
| 3 or more years older | 64 | 24 (38) | 1.32 (0.41 to 4.26) |  | 1.07 (0.32 to 3.67) |  |
|  |  |  |  |  |  |  |

BV=Bacterial vaginosis (Nugent score ≥7). SES=Socio-economic status. ^a^ Sensitivity analysis assuming participants with Y-chromosome and STIs (including TV, HPV and HIV) have all had penile-vaginal sex irrespective of self-reported sexual history. ^b^ SES indicator was fitted as a continuous covariate; the odds ratio of 0.70 estimates the decrease in odds of BV for a one-step increase in SES score. ^c^ Missing data for some participants. ^d^ Analysis restricted to those who reported having had at least one partner. ^e^ All were adjusted for age and SES; behavioural factors were also adjusted for lifetime sexual partners and oral sex.

| Table S2.2. Second sensitivity analysis of BV and associations with sexually transmitted infections among adolescent schoolgirls in Mwanza city, Tanzania.^a^ | | | | | | |
| --- | --- | --- | --- | --- | --- | --- |
|  | **N** | **BV**  **n (%)** | **Odds ratio**  **(95% CI)** | **p** | **Adjusted odds ratio (95% CI)** ^c^ | **p** |
| **Total** | 386 | 95 (25) | - | - | - | - |
| **Vaginal yeast** |  |  |  |  |  |  |
| Negative | 365 | 92 (25) | 1 | 0.268 | 1 | 0.291 |
| Positive | 21 | 3 (14) | 0.50 (0.14 to 1.72) |  | 0.50 (0.14 to 1.80) |  |
| **Chlamydia or gonorrhoea** |  |  |  |  |  |  |
| Negative | 369 | 91 (25) | 1 | 0.916 | 1 | 0.508 |
| Positive | 17 | 4 (24) | 0.94 (0.30 to 2.96) |  | 0.66 (0.20 to 2.23) |  |
| **Trichomonas vaginalis** |  |  |  |  |  |  |
| Negative | 369 | 89 (24) | 1 | 0.300 | 1 | 0.905 |
| Positive | 17 | 6 (35) | 1.72 (0.62 to 4.78) |  | 0.94 (0.31 to 2.82) |  |
| **Human papillomavirus** ^b^ |  |  |  |  |  |  |
| Negative | 259 | 50 (19) | 1 | <0.001 | 1 | 0.292 |
| Positive | 125 | 45 (36) | 2.35 (1.46 to 3.79) |  | 1.39 (0.75 to 2.57) |  |

BV=Bacterial vaginosis (Nugent score ≥7). ^a^ Sensitivity analysis assuming participants with Y-chromosome and STIs (including TV, HPV and HIV) have all had penile-vaginal sex irrespective of self-reported sexual history. ^a^ Data missing for two participants in the BV negative group. ^c^ All adjusted for age, SES, lifetime sexual partners, oral sex and HPV.
